# Physics of pure and non-pure positron emitters for PET: a review and a discussion

**DOI:** 10.1186/s40658-016-0144-5

**Published:** 2016-05-23

**Authors:** Maurizio Conti, Lars Eriksson

**Affiliations:** Siemens Healthcare Molecular Imaging, Knoxville, TN USA; Department of Physics, University of Stockholm, Stockholm, Sweden; Karolinska Institute, Stockholm, Sweden; Scintillation Material Research Center, University of Tennessee, Knoxville, TN USA

**Keywords:** PET, Radioisotopes, Positron emitter, Prompt gamma, Non-conventional PET isotopes

## Abstract

With the increased interest in new PET tracers, gene-targeted therapy, immunoPET, and theranostics, other radioisotopes will be increasingly used in clinical PET scanners, in addition to ^18^F. Some of the most interesting radioisotopes with prospective use in the new fields are not pure short-range β^+^ emitters but can be associated with gamma emissions in coincidence with the annihilation radiation (prompt gamma), gamma-gamma cascades, intense Bremsstrahlung radiation, high-energy positrons that may escape out of the patient skin, and high-energy gamma rays that result in some *e*^+^/*e*^−^ pair production. The high level of sophistication in data correction and excellent quantitative accuracy that has been reached for ^18^F in recent years can be questioned by these effects. In this work, we review the physics and the scientific literature and evaluate the effect of these additional phenomena on the PET data for each of a series of radioisotopes: ^11^C, ^13^N, ^15^O, ^18^F, ^64^Cu, ^68^Ga, ^76^Br, ^82^Rb, ^86^Y, ^89^Zr, ^90^Y, and ^124^I. In particular, we discuss the present complications arising from the prompt gammas, and we review the scientific literature on prompt gamma correction. For some of the radioisotopes considered in this work, prompt gamma correction is definitely needed to assure acceptable image quality, and several approaches have been proposed in recent years. Bremsstrahlung photons and ^176^Lu background were also evaluated.

**Electronic supplementary material:**

The online version of this article (doi:10.1186/s40658-016-0144-5) contains supplementary material, which is available to authorized users.

## Introduction

With the rise of interest in new PET tracers, multiple-tracer PET imaging, gene-targeted therapy, theranostics, and immunoPET [1–3], other radioisotopes are expected to be increasingly used in clinical PET scanners, side by side with ^18^F. Some short-lived radioisotopes have been extensively used in the past in the clinic or in research: ^15^O and ^11^C for brain studies, ^13^N and ^82^Rb for cardiac studies, and ^18^F and ^11^C for several applications in oncology. ^68^Ga use is showing a dramatic growth because of the applicability in labeling both small compound and macromolecules and because it is obtained from a relatively inexpensive ^68^Ge/^68^Ga generator system [4].

The use of monoclonal antibodies (mAb) as therapeutic target-specific agents has generated an interest in PET labeling of mAb [5, 6]. In order to better match the slower pharmacokinetics of the mAb, radioisotopes with a long half-life, such as ^64^Cu, ^76^Br, ^86^Y, ^89^Zr, and ^124^I, are more apt to follow the tracer and the radiopharmaceutical for hours, days, or even weeks [7, 8].

Moreover, the same radioisotopes are particularly suitable for the development of molecular imaging in tandem with a therapeutic agent, pharmaceutical or radiotherapeutic (also known as theranostics), for personalized treatment planning or real-time therapy monitoring. For example, ^86^Y and ^124^I can be used as a surrogate of ^90^Y and ^131^I, β^−^ emitters commonly adopted in radiotherapy, in order to accurately estimate the most likely dose distribution before radiotherapy [9]. Recently, it has been shown that ^90^Y can simultaneously work as an imaging agent and a therapeutic agent [10].

Finally, PET imaging with multiple tracers could be instrumental to better understand the molecular basis of a disease: multiple aspects of the disease can be characterized in the same study, and this would also allow for avoiding multiple scans, with cost reduction and improved patient comfort [11]. PET radioisotopes could be identified by different half-lives and also by their different decay mechanisms. In particular, a compound labeled with a pure positron emitter, as ^18^F, can be used together with another compound labeled with a radioisotope with a prompt gamma in coincidence with the positron decay, as ^124^I or ^86^Y: using the differential count rates of dual and triple coincidences can allow for differentiating and reconstructing the individual radioisotope activities [11–13].

Some of the most interesting radioisotopes with prospective use in the new fields are not pure short-range β^+^ emitters but can be associated with gamma emissions in coincidence with the annihilation radiation (prompt gamma), gamma-gamma cascades, intense Bremsstrahlung radiation, high-energy positrons that may escape out of the patient skin, and high-energy gamma rays that result in some *e*^+^/*e*^−^ pair production. The high level of sophistication in data correction and excellent quantitative accuracy that has been reached for ^18^F in recent years can be questioned by these effects. In this work, we review the physics and the scientific literature and evaluate the effect of these additional phenomena on the PET data for each of a series of radioisotopes: ^11^C, ^13^N, ^15^O, ^18^F, ^64^Cu, ^68^Ga, ^76^Br, ^82^Rb, ^86^Y, ^89^Zr, ^90^Y, and ^124^I. In addition, some experimental results are analyzed, using data from work previously published, or data ad hoc acquired for this article, in order to characterize the different phenomena.

The following effects will be discussed:

1. Prompt gamma emitted in coincidence with β^+^, within the energy window of the PET scanner
2. High-energy prompt gammas that fall within the energy window of the scanner after scatter in the patient
3. High-energy gammas generating *e*^+^/*e*^−^ pair production in the tissue or in the high *Z* detectors
4. High-energy positrons escaping the patient skin and annihilating in the scanner tunnel
5. Bremsstrahlung radiation
6. Radiation and coincidences from ^176^Lu or other radioactive impurities in the scintillator

## Review

The decay schemes of the radioisotopes of interest have been studied and are available through several reference web sites [14–16]. These are presented in simplified form in the Additional file 1 as a convenient summary for the reader. Radioisotope half-life, branching ratio, and photon and positron energies, reported in this article in the “Pure PET radioisotopes” and “PET radioisotopes with relevant contribution of prompt gammas” sections, are from the same sources. The positron ranges in water, as a surrogate of human tissue, are from the National Institute of Standards and Technology web site [14].

In this work, we focus on the aspects that could generate a viable PET signal. A few parameters can drive the use and success of a PET radioisotope:

1. Short or long half-life: the former is typically convenient for high-statistics PET scan of compounds that reach their target very quickly, like ^15^O in brain studies or ^18^F-FDG for oncology; the latter is used where the target is reached more slowly, as in ^89^Zr-labeled or ^64^Cu-labeled antibody imaging.
2. Energy of the positron: the energy determines the range in the tissue. Better spatial resolution can be reached with short-range positrons; ^11^C, ^18^F, ^64^Cu, and ^89^Zr are typical short-range positron emitters, and ^13^N, ^15^O, ^68^Ga, ^82^Rb, and ^124^I are long-range positron emitters (>1 mm).
3. Prompt gamma contamination: gammas emitted in decay cascades with the positron emission produce spurious coincidences, which blur the PET image and produce quantification errors, as in ^76^Br, ^86^Y, ^82^Rb, and ^124^I.
4. Emission of high-energy β^−^ decay: some radioisotopes have an alternative high-energy β^−^ decay that could be used for radiotherapy, and the minority β^+^ decay could be used for online monitoring or dosimetry of the radiotherapy, as in ^64^Cu or ^90^Y; others have a homologous radioisotope used for radiotherapy, and the β^+^ emitter can be used for treatment planning of the β^−^ emitter, as in ^131^I (β^−^ emitter) and ^124^I (β^+^ emitter).

For the sake of simplicity, we will organize the radioisotopes in terms of pure positron emitter and prompt gamma positron emitters. A special class will be dedicated to ^90^Y. A preliminary section is dedicated to a short review of the effect of positron range on spatial resolution and methods to correct for this effect.

### Effect of positron range on spatial resolution

Many of the PET radioisotopes suitable for immunoPET and theranostics emit positron with long positron range. This implies a blurring of the source distribution and a consequent loss of spatial resolution [17–19]. The loss of spatial resolution, compared to short-range positron emitter such as ^18^F, has been measured in high-resolution PET scanners for brain imaging and animal PET [20, 21]. For the present generation of commercial whole-body PET scanners, with spatial resolution of about 4 mm, the blurring due to positron range is barely measurable [22]. Nevertheless, in the future, even whole-body PET, with improved spatial resolution, might require a mechanism to recover the loss of resolution associated with long positron range.

An accurate characterization of the point spread function (PSF) allows for compensation of the loss in contrast recovery using calculated spill-over and partial volume corrections [23]. Methods to correct for positron range have been presented, as deblurring techniques [24] or incorporating positron range in the reconstruction itself [25]. Other researchers used the Monte Carlo simulation to estimate the distance between the source position and the detection line of response in the PET scanner and then incorporated this information in the reconstruction [19, 26]. The increasing use of these long-positron-range radioisotopes will make methods for resolution recovery even more necessary.

In addition, the long positron range of some of these radioisotopes can not only blur the primary radioisotope distribution but can also generate image artifacts. The positrons can exit the target organs and travel in air annihilating in a location where there is no tracer uptake, generating “ghost” uptake. This phenomenon has been observed in the trachea and in the larynx when ^124^I and ^68^Ga are used [27, 28], but it could also be present in low-density organs such as the lungs, especially in PET/MR systems where the positrons may follow the direction of the magnetic field.

### Pure PET radioisotopes

The most commonly used PET radioisotopes, such as ^11^C, ^13^N, ^15^O, and ^18^F, have short half-lives and high branching ratios for β+ decay. ^89^Zr has a long half-life, low branching ratio for β+ decay, and moderate-range positron emission, feeding an excited state with such a long half-life that the associated gamma ray from this excited level is not in coincidence with the PET signal. ^64^Cu has a long half-life, low branching ratio for β+ decay, short-range positron emission, and an additional decay branch via β^−^ emission. The main properties of these radioisotopes are summarized in Table 1.

**Table 1:** Properties of pure positron emission radioisotopes. Data from the National Institute of Standards and Technology [14], Laboratoire National Henri Becquerel [15], and Brookhaven National Laboratory [16]. Range of positrons is in water [14]

Isotope	Half-life	Branching (β^+^) in %	*E*_max_ (MeV)	*E*_mean_ (MeV)	*R*_max_ (mm)	*R*_mean_ (mm)
^11^C	20.4 min	99.8	0.960	0.386	4.2	1.2
^13^N	10.0 min	99.8	1.199	0.492	5.5	1.8
^15^O	2 min	99.9	1.732	0.735	8.4	3.0
^18^F	110 min	96.9	0.634	0.250	2.4	0.6
^64^Cu	12.7 h	17.5	0.653	0.278	2.5	0.7
^89^Zr	78.4 h	22.7	0.902	0.396	3.8	1.3

^11^C decays into ^11^B with a half-life *t*_1/2_ = 20.4 min. The decay is 99.8 % by β^+^ and 0.2 % by electron capture (EC, no radiation emitted). The emitted positron has 0.960-MeV maximum energy (*E*_max_) and 0.386-MeV mean energy (*E*_mean_), which corresponds to a 4.2-mm maximum range in water (*R*_max_) and a 1.2-mm mean range in water (*R*_mean_).

^13^N decays into ^13^C with a half-life *t*_1/2_ = 10.0 min. The decay is 99.8 % by β^+^ and 0.2 % by EC (no radiation emitted). The emitted positron has *E*_max_ = 1.199 MeV and *E*_mean_ = 0.492 MeV, which corresponds to a *R*_max_ = 5.5 mm and a *R*_mean_ = 1.8 mm.

^15^O decays into ^15^N with a half-life *t*_1/2_ = 2.0 min. The decay is 99.9 % by β^+^ and 0.1 % by EC (no radiation emitted). The emitted positron has *E*_max_ = 1.732 MeV and *E*_mean_ = 0.735 MeV, which corresponds to a *R*_max_ = 8.4 mm and a *R*_mean_ = 3.0 mm.

^18^F decays into ^18^O with a half-life *t*_1/2_ = 110 min. The decay is 96.9 % by β^+^ and 3.1 % by EC (no radiation emitted). The emitted positron has *E*_max_ = 0.634 MeV and *E*_mean_ = 0.250 MeV, which corresponds to a *R*_max_ = 2.4 mm and a *R*_mean_ = 0.6 mm.

^64^Cu decays, with a half-life *t*_1/2_ = 12.7 h, either β^−^ into ^64^Zn (38.5 %, with *E*_max_ = 0.579 MeV) or into ^64^Ni (61.5 %) via a combination of EC and β^+^. The EC has a branching ratio of 43.5 %, while the β^+^ decay is 17.5 %. The emitted positron has *E*_max_ = 0.653 MeV and *E*_mean_ = 0.278 MeV, which corresponds to a *R*_max_ = 2.5 mm and a *R*_mean_ = 0.7 mm.

^89^Zr decays into a metastable-state ^89m^Y, with a half-life *t*_1/2_ = 78.4 h. The decay is 22.7 % by β^+^ and 76.2 % by EC. The rest (1.1 %) of ^89^Zr decays by EC into several excited levels of ^89^Y, followed by emission of γs into the same metastable-level ^89m^Y. The emitted positron has *E*_max_ = 0.902 MeV and *E*_mean_ = 0.396 MeV, which corresponds to a *R*_max_ = 3.8 mm and a *R*_mean_ = 1.3 mm. The metastable-level ^89m^Y decays (*t*_1/2_ = 16 s) to a ground-state ^89^Y by emission of a γ (0.909 MeV). Because of the long half-life of this metastable-level ^89m^Y, the γ is in cascade with β^+^ but cannot be considered in coincidence and it does not behave as a prompt gamma.

### PET radioisotopes with relevant contribution of prompt gammas

The isotopes ^68^Ga, ^76^Br, ^82^Rb, ^86^Y, and ^124^I have complex decay schemes with a variety of gammas in coincidences. Some of them emit prompt gammas that directly fall into the energy window accepted by the PET scanner; others emit high-energy prompt gammas that have some probability of generating spurious coincidences after scattering in the patient or via *e*^+^/*e*^−^ pair production in the detector or the patient. The main properties of these radioisotopes are summarized in Table 2.

**Table 2:** Properties of prompt gamma positron emission radioisotopes. Only the positrons and prompt gammas with the two highest branching ratios are listed. Data from the National Institute of Standards and Technology [14], Laboratoire National Henri Becquerel [15], and Brookhaven National Laboratory [16]

Isotope	Half-life	Branching (β^+^) in %	β^+^ *E*_max_ (MeV)	Branching (γ) in %	γ E (PG) (MeV)
^68^Ga	67.8 min	87.7, 1.2	1.899, 0.821	3.2	1.077
^76^Br	16.2 h	25.8, 6.3	3.382, 0.871	74.0, 15.9	0.559, 0.657
^82^Rb	1.3 min	81.8, 13.1	3.378, 2.601	15.1	0.777
^86^Y	14.7 h	11.9, 5.6	1.221, 1.545	82.5, 32.6	1.077, 0.627
^124^I	100.2 h	11.7, 10.7	1.535, 2.138	62.9, 11.2	0.602, 1.691

^68^Ga decays into ^68^Zn, with a half-life *t*_1/2_ = 67.8 min. The decay is 88.9 % by β^+^ and 11.1 % by EC. The main β_1_^+^ decay (87.7 %) is to the ground level of ^68^Zn (*E*_max_ = 1.899 MeV, *E*_mean_ = 0.836 MeV, *R*_max_ = 9.2 mm, *R*_mean_ = 3.5 mm), and it is a pure positron emission branch. A small fraction decays β_2_^+^ (1.2 %) into an excited level of ^68^Zn, which promptly (*t*_1/2_ = 1.6 ps) decays into the ground level with a γ_1_ (1.077 MeV). This can constitute a small prompt gamma contamination in the PET data, if the 1.077-MeV γ_1_ has a scatter interaction in the patient, and generates a detectable lower energy γ in coincidence with the positron annihilation pair. The principal EC branches are to the ground level (8.9 %) or to the first excited level (1.8 %). The rest of the EC (0.4 %) decays are into short-life excited levels emitting γs of various energies (0.227–2.821 MeV), with negligible probability to be detected in coincidence.

^76^Br decays into ^76^Se, with a half-life *t*_1/2_ = 16.2 h. The decay is 54.8 % by β^+^ with different energies and 45.2 % by EC. The only pure positron emitter decay β_3_^+^ decays to the ground level of ^76^Se with a branching ratio of only 6.0 %. The other major β^+^ decay modes populate an excited level of ^76^Se, with emission of cascades of prompt gammas: β_1_^+^, β_2_^+^, and β_4_^+^ (25.8, 6.3, and 5.2 %, respectively) and a large number of other β^+^ decays that add up to 11.5 %. Most β^+^s are in cascade with a prompt gamma γ_1_, with energy (0.559 MeV) very close to the typical PET energy window. In summary, about 90 % of ^76^Br positrons are associated with one or more prompt gammas. A large fraction of prompt gammas has energy within the PET energy window; a smaller fraction of the high-energy gammas could scatter in the patient and produce photons also within the PET energy window; and others can directly generate *e*^+^/*e*^−^ pairs. The energy of the β^+^ is typically high, with a long range and resulting in poor spatial resolution.

^82^Rb decays into ^82^Kr, with a half-life *t*_1/2_ = 1.3 min. The decay is β^+^ with two main energies. The pure positron decay β_1_^+^ is to the ground level of ^82^Kr, with a branching ratio of 81.8 % (*E*_max_ = 3.378 MeV, *E*_mean_ = 1.535 MeV, *R*_max_ = 17.0 mm, *R*_mean_ = 7.1 mm). The second most frequent positron decay β_2_^+^ is to an excited level of ^82^Kr, with a branching ratio of 13.1 % (*E*_max_ = 2.601 MeV, *E*_mean_ = 1.168 MeV, *R*_max_ = 13.0 mm, *R*_mean_ = 5.0 mm). As a consequence, a prompt gamma γ_1_, with energy 0.777 MeV, is emitted in cascade, generating prompt gamma coincidences, mainly after scatter in the patient body. Both β_1_^+^ and β_2_^+^ have high energy and consequently long ranges.

^86^Y decays into ^86^Sr, with a half-life *t*_1/2_ = 14.7 h. The decay is 31.9 % by β^+^ with different energies and 68.1 % by EC. All β^+^ decay modes populate excited levels of ^86^Sr, with emission of cascades of prompt gammas. The main β^+^ decay modes are β_1_^+^ (11.9 %, *E*_max_ = 1.221 MeV, *E*_mean_ = 0.535 MeV, *R*_max_ = 5.6 mm, *R*_mean_ = 1.9 mm), in cascade with γ_5_ (1.920 MeV) and γ_1_ (1.077 MeV); β_2_^+^ (5.6 %, *E*_max_ = 1.545 MeV, *E*_mean_ = 0.681 MeV, *R*_max_ = 7.1 mm, *R*_mean_ = 2.8 mm), in cascade with γ_7_ (0.443 MeV), γ_3_ (1.153 MeV), and γ_1_ (1.077 MeV); and β_3_^+^ (3.6 %, *E*_max_ = 1.988 MeV, *E*_mean_ = 0.883 MeV, *R*_max_ = 9.7 mm, *R*_mean_ = 3.7 mm), in cascade with γ_3_ (1.153 MeV) and γ_1_ (1.077 MeV). Even non-β^+^ decays generate cascades of gammas in coincidence. All gamma cascades can generate spurious coincidences: some prompt gammas have energy within the PET energy window. Part of the high-energy gammas could scatter in the patient and produce photons also within the PET energy window, and others can directly generate *e*^+^/*e*^−^ pairs. The energy of the β^+^ is typically high, with a long range and with degraded spatial resolution.

^124^I decays into ^124^Te, with a half-life *t*_1/2_ = 100.2 h. The decay is 77.3 % by EC and 22.7 % by β^+^ with two main modes: a pure positron decay to the ground level of ^124^Te, β_2_^+^ (10.7 %, *E*_max_ = 2.138 MeV, *E*_mean_ = 0.975 MeV, *R*_max_ = 10.0 mm, *R*_mean_ = 4.4 mm), and a β_1_^+^ mode (11.7 %, *E*_max_ = 1.535 MeV, *E*_mean_ = 0.687 MeV, *R*_max_ = 7.1 mm, *R*_mean_ = 2.8 mm), which decays into an exited state of ^124^Te, with a prompt gamma emission γ_1_ (0.602 MeV). There is an additional β_3_^+^ (0.3 %, *E*_max_ = 0.812 MeV, *E*_mean_ = 0.367 MeV, *R*_max_ = 3.4 mm, *R*_mean_ = 1.1 mm), with a prompt gamma emission γ_3_ (0.723 MeV), also in cascade with emission γ_1_ (0.602 MeV). Moreover, a large part of the excited level is populated by EC decays via gamma cascades into the first excited level of ^124^Te that decays into the ground level with the same γ_1_ (602 MeV), and they can produce coincidences between γ_1_ and photons in the cascade. Some gammas have energy within the PET energy window. A fraction of the high-energy gammas could undergo Compton scatter in the tissue, and the scattered photons might enter the PET energy window. Other high-energy gammas can directly generate *e*^+^/*e*^−^ pairs. The energies of the β^+^ are high, associated with a long range and poor spatial resolution.

### ^90^Y, a non-β^+^ decay PET radioisotope

^90^Y is mainly a β^−^ emitter used for radiotherapy and associated with very high Bremsstrahlung radiation but has a very small branching ratio that produces *e*^+^/*e*^−^ pairs, a weak but viable PET signal [10].

^90^Y decays (*t*_1/2_ = 64.1 h) 99.99 % into the ground-level ^90^Zr by β_1_^−^ (2.280 MeV) and 0.0115 % into an excited level of ^90^Zr by β_2_^−^ (0.519 MeV). The high-energy β_1_^−^ is used for radiotherapy, and the fast electrons of β_1_^−^ emit strong Bremsstrahlung radiation. The excited level associated with β_2_^−^ decays into a ground state of ^90^Zr by internal conversion (0.0083 %) or creation of an *e*^+^/*e*^-^ pair (0.0032 %) [29]. This small branching ratio in the *e*^+^/*e*^-^ pair annihilates and can be observed as a PET signal. The *e*^+^/*e*^-^ pair can have a maximum total kinetic energy of 0.739 MeV; therefore, it has a short range.

### Prompt gamma characterization

PET data reconstruction is based on a two-step process: estimating the corrections to the data and image reconstruction itself. The data corrections are normalization (individual detector sensitivity, sensitivity profile differences due to radial position of the source, and other factors relative to the scanner geometry), attenuation correction (due to the interaction of emitted photons with the patient tissue), random correction (accidental coincidence of uncorrelated photons at a high count rate), and scatter correction (photons having a Compton interaction in the patient and therefore changing energy and direction). Typically, all corrections assume that only two back-to-back 511-keV photons from positron annihilation are involved: individual detector sensitivity is measured with 511-keV photons, attenuation correction is measured with CT X-rays but is converted into a 511-keV attenuation coefficient, and scatter is simulated or estimated based on single (or multiple) scatter simulations or models of interaction of 511-keV photons. Randoms are usually estimated using a delayed window or from single models, measuring single count rates in detector. Because of this, the random estimate does not include any assumption about the energy or the direction of the detected photons, and a random estimate method can work with any radioisotope.

Most clinical applications of PET require ^18^F-FDG PET imaging. Tracers based on ^18^F are pure β^+^ emitters, and only 511 keV is emitted. Moreover, ^18^F’s positrons have a short range (<1 mm), and the position of the radioisotope can be approximated with the position of the positron annihilation. As we discussed in the previous section, also ^11^C, ^13^N, and ^15^O are pure positron emitters. Because of the longer range of ^13^N and ^15^O, those radioisotopes might result in slightly blurred images or require positron range deblurring. ^64^Cu and ^89^Zr can be considered pure β^+^ emitters, even though they have a much lower branching ratio: ^64^Cu decays β^+^ to the ground level of ^64^Ni, and ^89^Zr decays β^+^ to a metastable level of Yttrium ^89m^Y, but the long half-life of the metastable level (15.8 s) makes the associated gamma not in coincidence.

**Fig. 1:**
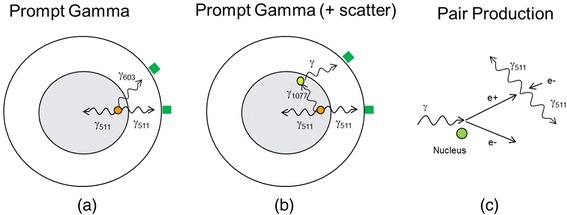
Three mechanisms associated with prompt gamma spurious coincidences: **a** ^124^I has a prompt gamma of energy within or close to the PET energy window (603 keV); **b** ^68^Ga has a prompt gamma of energy that can enter the PET energy window if it undergoes Compton scatter (1077 keV); and **c** ^76^Br has several high-energy gammas that can reach the detectors and generate *e* ^+^/*e* ^−^ pairs

If the decay scheme is more complex and involves additional radiation in coincidence, then the approximations for pure β^+^ emitters do not hold well, and the data correction is more difficult, as in the case of ^68^Ga, ^76^Br, ^82^Rb, ^86^Y, and ^124^I. Spurious coincidences can be produced by prompt photons or high-energy photons generated during the decay of the injected radioisotope (Fig. 1). A prompt gamma can be detected because it has energy within or close to the PET energy window (Fig. 1a) or after it undergoes Compton scatter that reduces its energy (Fig. 1b). Moreover, high-energy gammas—in cascade with a β^+^ or β^−^ decay or produced by another decay mechanism—can reach the detectors and generate *e*^+^/*e*^−^ pairs (Fig. 1c). In this case, their energy must be over the threshold of 1.022 MeV, and the pair production probability increases with the photon energy. Also, in some decay modes, high-energy levels are populated via EC, and they decay to the ground level in cascade of two or more gammas. These cascades add to the background of spurious coincidences.

Finally, radioisotopes with a prompt gamma can generate not only coincidences between two photons but also triple coincidences, between two annihilation gammas and a prompt gamma. The fraction of triple coincidences has been estimated to be about 25 % for ^124^I and greater than 50 % for ^86^Y [30]. The different ratios of double and triple coincidences in pure beta emitter and non-pure beta emitter have been exploited as a method to differentiate radioisotopes in multiple-tracer imaging [12, 13].

**Fig. 2:**
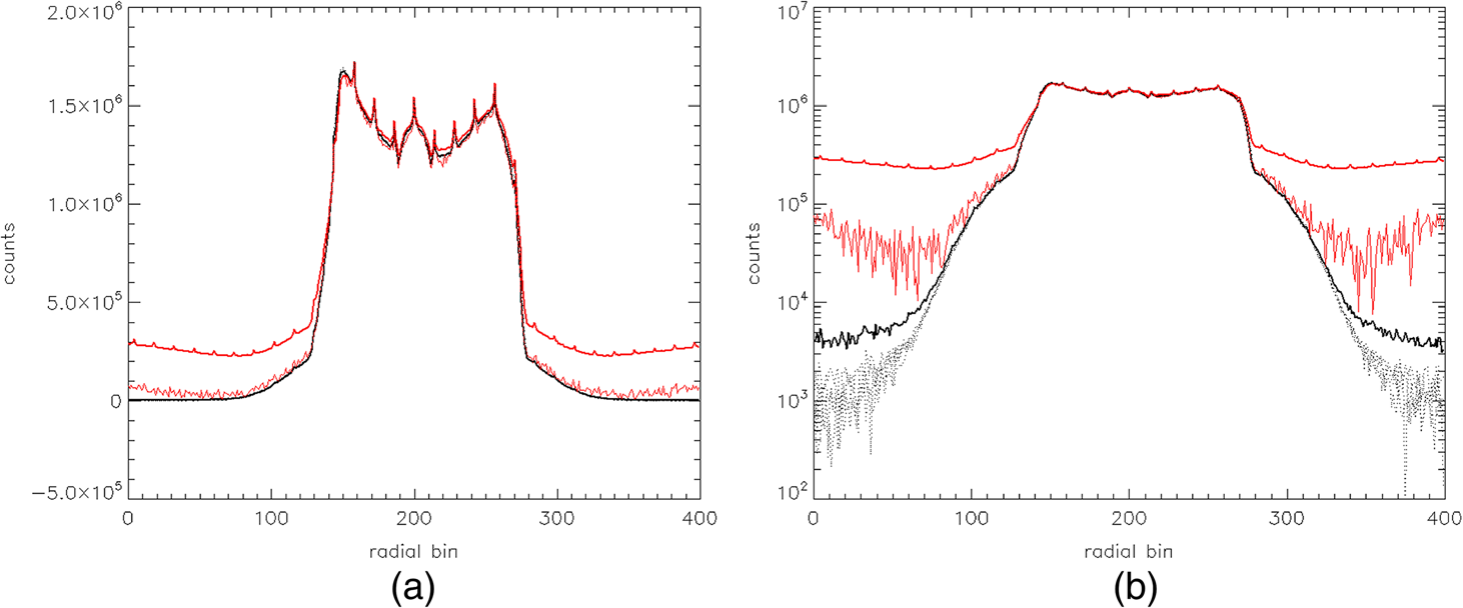
Radial net true sinogram profiles (all angles added) of a NEMA image quality phantom, filled with water solutions of several radioisotopes used for PET imaging: ^68^Ga (*thick black line*), ^90^Y (*thin red line*), ^124^I (*thick red line*), and ^11^C, ^18^F, ^64^Cu, and ^89^Zr (all in *black dotted lines*). The phantom was scanned on a Siemens mCT PET/CT scanner. The profile is shown after normalization to maximum, **a** in linear scale and **b** log scale

The contribution of the prompt gamma coincidences has a different spatial distribution when compared to the pure annihilation coincidences. This can be observed by looking at the radial profiles of sinograms obtained using the same phantom but with different radioisotopes. The data, reprocessed for this article, were acquired during an experiment aimed to characterize PET scanner performance, and details are given in [22]. A NEMA image quality phantom was scanned in the same position several times and filled with water solutions of several radioisotopes used for PET imaging: ^11^C, ^18^F, ^64^Cu, ^68^Ga, ^89^Zr, ^90^Y, and ^124^I. The phantom was scanned on a Siemens mCT PET/CT scanner, and 200 million net true counts, defined as prompt coincidences after delayed subtraction, were acquired for each experiment (only 10 million for ^90^Y). In Fig. 2, the radial profile is plotted for all the radioisotopes, after normalization to maximum value. One can observe that ^18^F, ^11^C, ^64^Cu, and ^89^Zr (pure β^+^ emitters) have very similar radial profiles; in fact, they fully overlap in the noisy regions of the plot. Also, the tails of the sinograms quickly decrease. ^68^Ga exhibits slightly higher tails, compared to the pure β^+^ emitters, which is evidence of a minor presence of prompt gammas. As expected, ^124^I has a large fraction of the coincidences that appear to have originated outside the phantom, a substantial contribution from prompt gamma coincidences. ^90^Y has intermediate behavior, with small but visible tails increasing towards the edges of the scanner.

In fact, ^68^Ga has only 1.2 % of β^+^ to an excited level, associated with a 1.077-MeV γ, compared to 87.7 % of β^+^ to the ground level. And the prompt gamma contribution to coincidences is even lower because the γ has to scatter and reduce its energy to be detected. Nevertheless, it has been observed that if the ^68^Ga activity is concentrated in a specific organ, and the scatter is evaluated away from the concentrated activity, this small background of the prompt gamma can create artifacts and generate scatter overestimate. In these occurrences, it has been recommended to use some prompt gamma correction even for ^68^Ga [31].

^82^Rb, used for cardiac studies, has a higher prevalence of the prompt gamma, 13.1 % of β^+^ to an excited level, associated with a 0.777-MeV γ, compared to 81.8 % of β^+^ to the ground level. The detection probability of the prompt gamma is reduced by the fact that only a small fraction of the 0.777-MeV γs falls into the energy window (typically, the upper threshold is between 550 and 650 keV) and another fraction enters the window only after scattering in the tissue. In any case, a prompt gamma correction is absolutely necessary for ^82^Rb.

For both ^82^Rb and ^68^Ga, a uniform radial offset or a first-order polynomial well describe the prompt gamma, probably because the scattered detected gammas have quite a flat spatial distribution.

**Fig. 3:**
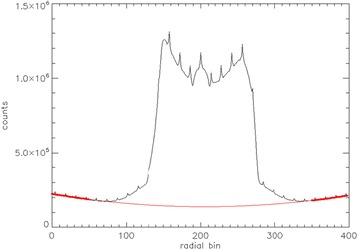
Radial net true sinogram profiles (all angles added) of a NEMA image quality phantom, filled with water solutions of ^124^I. A second-order polynomial is used to fit the background: the ^124^I profile (*black line*), the second-order polynomial fit (*red line*), and the tail region used for the fit (*thick red dotted line*) are shown. The phantom was scanned on a Siemens mCT PET/CT scanner

The contribution of the prompt gamma becomes prevalent in ^124^I, where 10.7 % are β^+^ decays to the ground level and 11.7 % are β^+^ decays to the excited level associated with a 0.603-MeV γ, fully within the PET energy window. Moreover, some of the remaining 77.3 % of the EC decays generated cascades of γs terminating in the same 0.603-MeV excited level, with possible additional spurious coincidences. In fact, we could roughly estimate the ratio of the prompt gamma over total coincidences in a sinogram. “True” coincidences (two 511-keV photons) are about 10.7 % + 1/3 11.7 % (considering a triple event with two 511-keV photons and one 603-keV photon). “Prompt gamma” coincidences (one 511-keV photon and one 603-keV photon) are about 2/3 11.7 %. This results in a ratio of the prompt gamma over total of about 0.35, a relevant fraction of the total coincidences. Figure 3 shows a second-order polynomial fit of the background in the image quality phantom sinogram; with this fit, we measured a 0.33 ratio of the prompt gamma over total coincidences. A second-order polynomial seems a better fit for ^124^I, due to the concavity of the background. One possible explanation for this shape is the fact that the 603-keV photons are directly detected into the energy window and the spatial distribution is dictated by the simple attenuation of the photons in the tissue [32].

Finally, ^76^Br and ^86^Y are dominated by decay schemes with prompt gammas. For ^76^Br, the largely dominant main β^+^ decay (25.8 %) is associated with a 0.559-MeV γ; in addition, other decay modes are characterized by γ cascades. In the ^86^Y, all the dominant β^+^ decays (11.9, 5.6, and 3.6 %) are associated with a 1.077-MeV γ; in addition, other decay modes terminate into the same 1.077-MeV level. Prompt gamma correction is absolutely necessary, since spurious coincidences are the majority of the coincidences for these radioisotopes.

### Prompt gamma correction

Standard scatter correction and random correction alone are not sufficient to obtain quantitative images in the presence of abundant spurious coincidences from prompt gammas. In the absence of proper correction, this results in high artifact background levels and consequent loss of contrast, or overestimation of the conventional scatter, with over subtraction in the central part of the body. In order to mitigate the effect of the prompt gamma, it has been shown that a narrower energy window can effectively reduce the prompt gamma contamination in the data [33]. This alone does not eliminate the problem, particularly in situations where the prompt gammas have energy very close to the conventional PET energy (511 keV) or the prompt gamma coincidences are the majority component. Moreover, it does reduce the sensitivity of the PET scanner to true coincidences. A new correction is needed, the prompt gamma correction (PGC). In the past years, several methods have been proposed to estimate and subtract the prompt gamma component.

The first group of methods consists of estimating the prompt gamma background fitting the radial tails of the sinogram with a constant offset and first- or second-order polynomials. At the radial extremities of the sinogram, no source of activity is present, the single or multiple scatter of 511 keV from positron annihilation is negligible. The offset due to prompt gammas can be evaluated and subtracted. Subtracting a simple uniform background has been shown to be effective even with ^86^Y and ^124^I, which have abundant prompt gamma fraction [34–36]. A linear approximation of the background has been used successfully for ^76^Br and proposed in general for all prompt gamma radioisotopes [37–39]. In order to better match the slightly concave shape of the background observed in ^86^Y and ^124^I [34], a second-order polynomial fit to the tails has been proposed (and tested on ^86^Y) [40].

A second approach is based on the observation that, for ^82^Rb, the prompt gamma background has a similar radial distribution as the randoms [41]. The authors proposed a method that uses the scaled random estimate, added to the single scatter conventional estimate, to match the tails of the prompt sinogram. The random scaling factor, obtained by fitting the tails, includes the prompt gamma contribution. This approach to prompt gamma correction was also used on ^124^I data, where the ^124^I image quality after PGC was comparable to that shown in a similar experiment with ^18^F [42].

A third approach is based on a convolution of the estimate of the original activity with some kernel based on models that include prompt gamma generation. One option uses a spatially variant, attenuation-dependent kernel that has been analytically determined and is based upon a simplified model of the cascade-coincidence attenuation and detection process [43]. The model is based on the measured attenuation map of the patient. In this method, the emission sinogram is preliminarily corrected with the standard corrections, then convolved with the kernel, and the tails of the convolved sinogram and the measured sinogram are fitted to obtain a scaling factor for the convolution kernel. Then, the scaled kernel can be used for the final estimate of the prompt gamma correction. This method has been tested with ^76^Br and ^86^Y data. Another group proposed a correction method for ^86^Y, based on the convolution of the estimate of the original activity with a ^86^Y point spread function kernel [44]. The resulting prompt gamma estimate is then normalized to counts outside the patient body, in order to obtain a more accurate correction. The method can be iterated. The ^86^Y point spread function kernel was measured once with an elliptical phantom that simulates the abdomen. Even with its limitation (the point spread function is based on a phantom), the results are encouraging.

A fourth approach is based on a simple attenuation model (SAM) for prompt gammas [32], analog to the single scatter model for single scatter simulation (SSS) [45]. This method assumes that the principal effect driving the spatial distribution of the prompt gamma coincidences in ^124^I is the simple attenuation of the 0.603 MeV in the patient body. The attenuation probability, for each source position and each detector, is computed geometrically, based on the expected travel path in the patient. As for the SSS, the original emission sinogram and the measured attenuation map of the patient are used to estimate a first approximation of the activity distribution. At each iteration, the attenuation of the prompt gammas is estimated via the new SAM method and the scatter of the annihilation photons via the conventional SSS. This method has shown excellent matching of the radial shape for ^124^I [32].

Finally, a possible but time-consuming solution is a full Monte Carlo simulation of the emission and interaction of all radiation in the prompt gamma radioisotopes. Fast-simulation packages for PET like SimSET have been used [46]. GATE Monte Carlo simulation was successful for a complete simulation of non-pure radioisotopes, and it was used as a support for estimating suitable functions modeling the tails of the sinograms. In particular, a two-component correction was proposed with the aid of GATE simulations, a uniform background and a Gaussian function [47, 48].

### Additional sources of spurious coincidences: positron range, Bremsstrahlung X-rays, LSO background

**Fig. 4:**
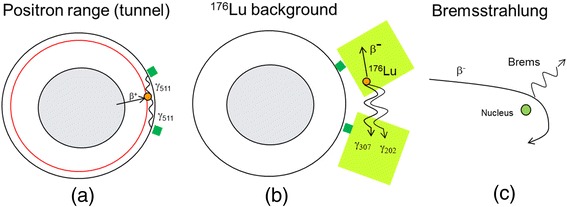
Additional mechanisms associated with spurious coincidences: **a** high-energy positrons, escaping the patient skin, can travel in air and reach the PET scanner internal tunnel, where they annihilate and produce two 511-keV gammas in coincidence; **b** in LSO- or LYSO-based PET scanners, a small background of ^176^Lu radiation can generate coincidences between β^−^ in a detector and two photons (307 keV + 202 keV) in another; and **c** high-energy Bremsstrahlung X-rays can be emitted by slowing down of high-energy β^−^ or β^+^ and create *e* ^+^/*e* ^−^ pairs in the detectors

Three additional phenomena can contribute to data contamination: high-energy positrons interacting with the scanner tunnel, background radiation in detectors such as lutetium orthosilicate (LSO) and lutetium yttrium orthosilicate (LYSO), and Bremsstrahlung X-rays emitted by slowing down of high-energy β^−^ or β^+^, which can create *e*^+^/*e*^−^ pairs in the detectors (Fig. 4). In this section, we will briefly discuss these effects.

High-energy positrons, associated with ranges larger than a few millimeters, can escape the patient skin. The positrons can travel in air and reach the PET scanner internal tunnel, where they annihilate and produce two 511-keV gammas in coincidence, completely uncorrelated with the distribution of the radioisotope in the patient (Fig. 4a). This effect might be present in β^+^ emitters with a high branching ratio for high-energy positrons: ^68^Ga (87.7 % of β^+^ with *R*_max_ = 9.2 mm and *R*_mean_ = 3.5 mm), ^76^Br (25.8 % of β^+^ with *R*_max_ = 17.4 mm and *R*_mean_ = 7.1 mm), ^82^Rb (81.8 % of β^+^ with *R*_max_ = 17.0 mm and *R*_mean_ = 7.1 mm), ^86^Y (with most of β^+^ with *R*_max_ between 5 and 15 mm), and ^124^I (11.7 % of β^+^ with *R*_max_ = 7.1 mm and *R*_mean_ = 2.8 mm and 10.7 % of β^+^ with *R*_max_ = 10.0 mm and *R*_mean_ = 4.4 mm).

**Fig. 5:**
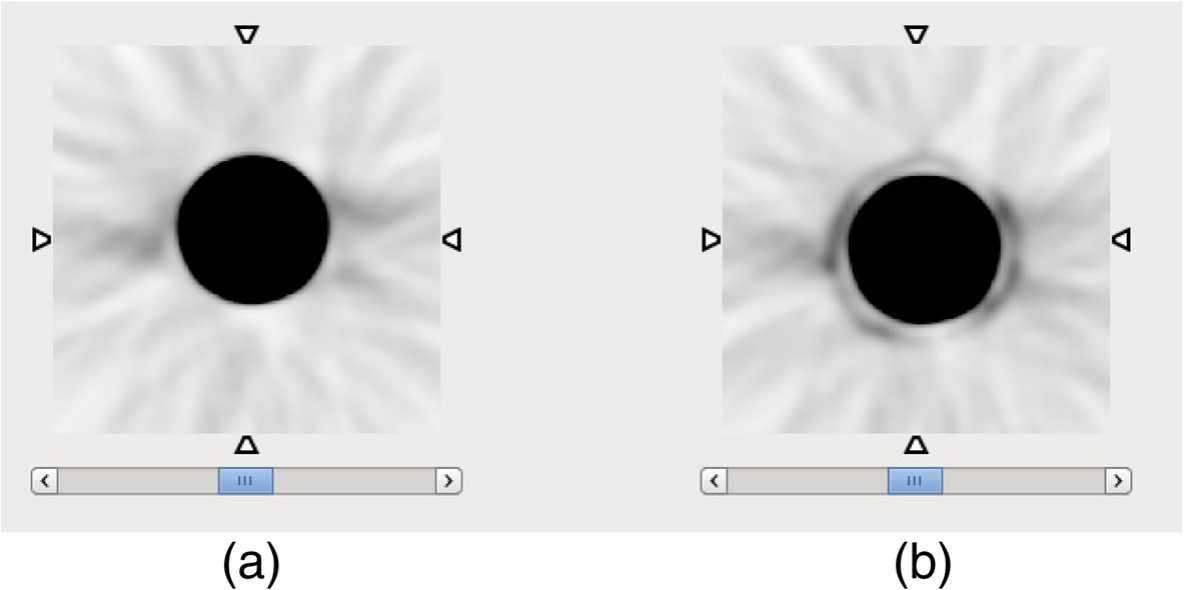
Transaxial planes of the reconstructed images of ^68^Ge/^68^Ga cylindrical source in the scanner, **a** without and **b** with a copper shield. The positrons escaping the phantom annihilate in the copper, which becomes a source of 511 gamma pairs, and are visible in the image as a shell surrounding the phantom

We have performed a simple experiment to visualize the β^+^ traveling in air, when the energy is high enough to escape the patient or the phantom. A 5.4-cm-diameter cylindrical ^68^Ge phantom was placed at the center of a Siemens mCT scanner, with and without a copper shield (0.64 mm). ^68^Ge disintegrates 100 % (half-life 270 days) by EC to a ^68^Ga ground state, and it is effectively a ^68^Ga source. The source external wall was 3 mm of plastic that has the density of water, which is not enough to stop β^+^ with *R*_max_ = 9.2 mm and *R*_mean_ = 3.5 mm. In Fig. 5a, a transaxial plane of the iterative reconstruction image of the activity distribution in the phantom without copper shield is shown. In Fig. 5b, the image of the phantom wrapped in copper foil is shown. One can observe the presence of the copper shield, stopping the positrons that otherwise would travel in air and reach the internal tunnel of the PET scanner. In the absence of the shielding, the spatial distribution of coincidence events is affected by the high-range positrons. In fact, similar effects have been observed in patient and phantom data, if ^124^I and ^68^Ga are used [27, 28].

**Fig. 6:**
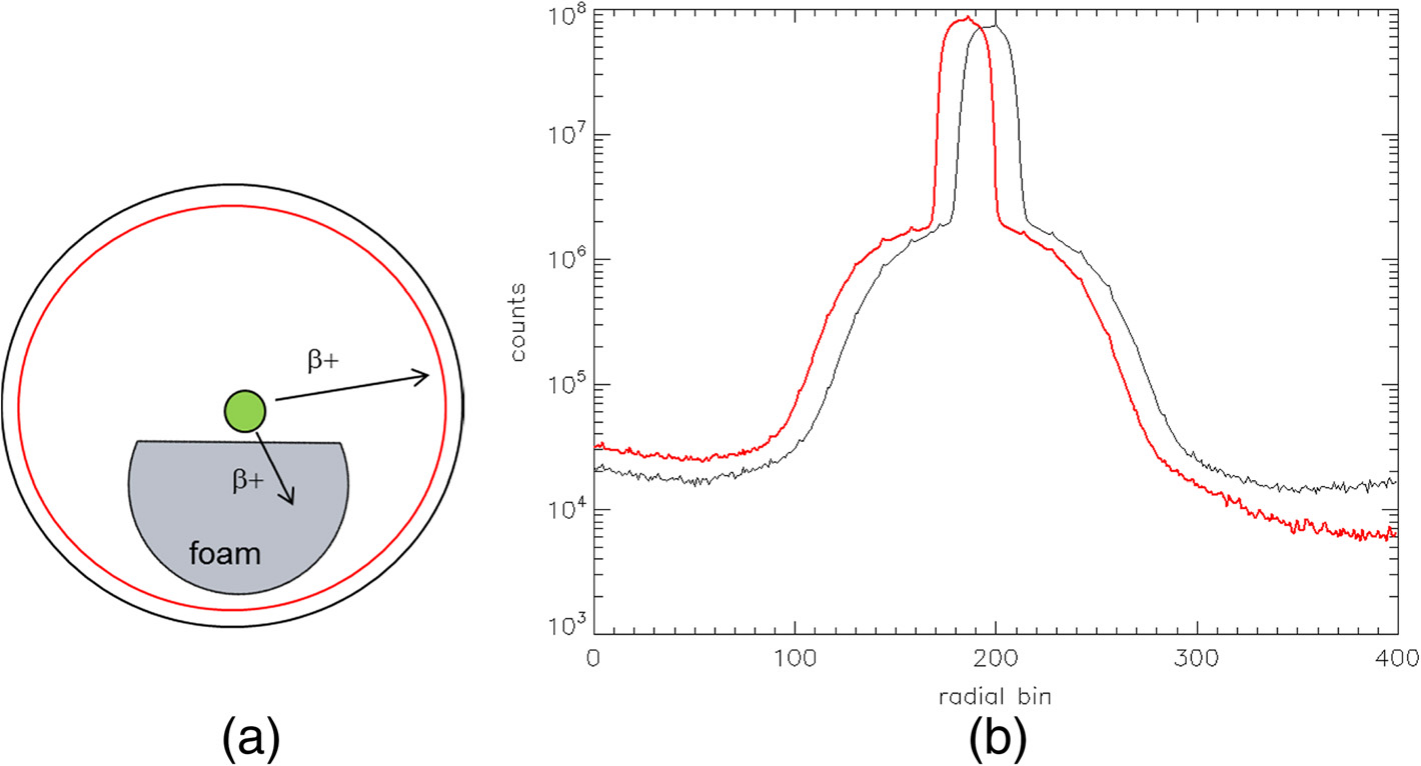
**a** Drawing of a ^68^Ge/^68^Ga phantom on a foam support inside a PET scanner tunnel. **b** Net true sinogram radial profile in the top-view projection, or at 0° (*black line*), and in the side-view projection, or 90° (*red line*)

The ^68^Ge source was held in place in the center of the field of view by a polymer foam holder covering the bottom part of the tunnel. The foam is unable to stop 511-keV photons but can reduce the flux of positrons reaching the lower part of the tunnel (Fig. 6a). At angle zero (top view), the radial distribution is fully symmetrical. An asymmetry can be observed at a 90° view (side view) (Fig. 6b): the right-side tail shows a drop of counts that is due to the suppression of the β^+^ flux reaching the tunnel. In fact, while this effect can be observed in phantoms, in patients, it might be very negligible. If the radiotracer is highly specific, the β^+^ emission is likely to be concentrated in the internal organs of interest, and the high attenuation will prevent the β^+^ from exiting the body. If the tracer is less specific and abundantly reaches the patient skin, this effect can appear.

In addition, sources of radioactivity completely independent of the patient can generate a background radiation: for example, in LSO- or LYSO-based PET scanners, a small background of ^176^Lu radiation can be seen when the PET signal in the patient is extremely low, as in ^90^Y imaging. LSO contains 2.6 % of ^176^Lu, a radioactive isotope that decays β^−^ into ^176^Hf with a cascade of γ photons in coincidence, with 307-, 202-, and 88-keV energies. A coincidence of a β^−^ in a detector and two photons (307 keV + 202 keV) in another is unlikely but possible (Fig. 4b). The ^176^Lu β^−^ in a detector generates about 5000 single counts per second per block or a total random counts of about 1100 s^−1^ in a four-ring Biograph mCT [49, 50]. This is a small but not negligible background for some applications at a very low count rate, such as with ^90^Y imaging. On the other hand, it has been demonstrated that randoms can be properly measured and corrected using standard random correction, even for ^90^Y imaging [50]. From the point of view of net true coincidence rate, after random subtraction, about 1 to 5 total count/s have been measured in the same four-ring LSO-based mCT when no source was present [49, 50].

**Fig. 7:**
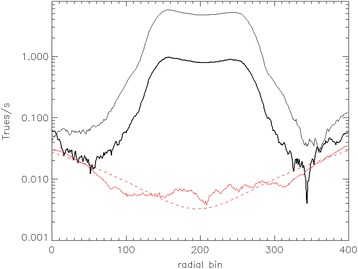
Net true sinogram radial profile of an image quality phantom with 3785 MBq ^90^Y (*thin black line*), 613 MBq ^90^Y (*thick black line*), background acquisition with no source (*red line*), and a polynomial fit of the background (*broken red line*)

The small contribution of LSO background in extremely low count scans can be appreciated in Fig. 7, where the sinogram profiles of true counts per second are shown for a ^90^Y image quality phantom. The data were acquired during an investigation aimed to characterize PET imaging of ^90^Y [50] and were re-analyzed for the present article. The upper black curve is a 30-min scan at 3.785 GBq, the lower black curve is a 30-min scan at 0.613 GBq, the red line represents a background measurement with no source, and the broken red line represents a second-order polynomial fit to the background. The background is 6.5 total net true/s. One can observe that at extremely low counts, the tails of the ^90^Y match the background measurement, while some residuals can be seen at the higher count rate, possibly due to residual scatter or pair production from high-energy Bremsstrahlung photons.

In the presence of very high activity, high-energy β^−^ or β^+^ can produce Bremsstrahlung radiation (Fig. 4c). Generally, a few thousands of MBq of ^90^Y are injected in the patients, and the 99.98 % of high-energy β^−^s from the decay produce a very high flux of Bremsstrahlung X-rays, which have a broad spectrum with endpoint 2.279 MeV. They can reach the detectors and produce *e*^+^/*e*^−^ pairs and therefore annihilation photons. Some authors propose this as an explanation for the low background tails as seen in Fig. 7 [51].

All the possible phenomena (LSO background, Bremsstrahlung X-rays) add up to a small net true contribution that still need to be properly considered for ^90^Y imaging, given the extremely low ^90^Y branching ratio of the *e*^+^/*e*^−^ pair production [52, 53]. Nevertheless, even with a background of singles, randoms, and net trues, LSO and LYSO scanners have been proven to function better than BGO-based scanners because of the time-of-flight capability associated with fast LSO/LYSO scintillators [53]. In general, the extremely small background of net true coincidences is neglected, and no additional correction, apart from standard scatter and random corrections, is performed for ^90^Y imaging in the commercial PET scanners.

## Conclusions

With the foreseeable growth of the use of PET beyond conventional ^18^F-FDG oncology, PET scanner performance needs to be characterized with a wider range of radioisotopes that are not all pure β^+^ emitters. In this article, we reviewed the physics of several most used or most promising radioisotopes, with focus on the features that can generate artifacts or challenges to the image reconstruction. In particular, we discussed the present complications arising from the so-called prompt gammas, and we reviewed the limited scientific literature on prompt gamma correction. The effect of high positron range, Bremsstrahlung photons, and ^176^Lu background were also evaluated.

## Additional file

Additional file 1: Decay schemes of radioisotopes for PET. (docx 1.79 MB)
