# Supplementary material for: Physics of pure and non-pure positron emitters for PET: a review and a discussion
Source: EJNMMI Phys. 2016 May 23;3:8. doi: 10.1186/s40658-016-0144-5 (PMC4894854; doi:10.1186/s40658-016-0144-5)
Supplement: Additional file 1: — Decay schemes of radioisotopes for PET. (docx 1.79 MB) [file 40658_2016_144_MOESM1_ESM.docx]

**APPENDIX: DECAY SCHEMES OF RADIOISOTOPES FOR PET**


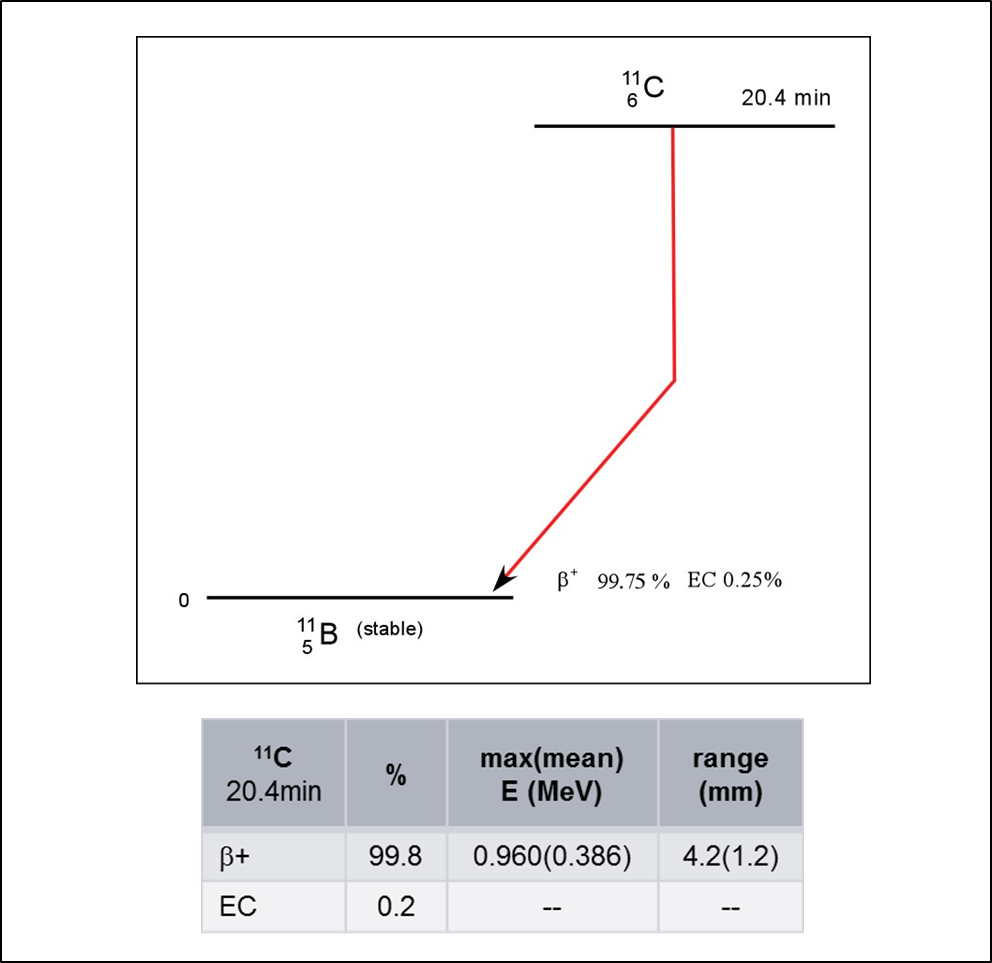


Figure A1: ^11^C


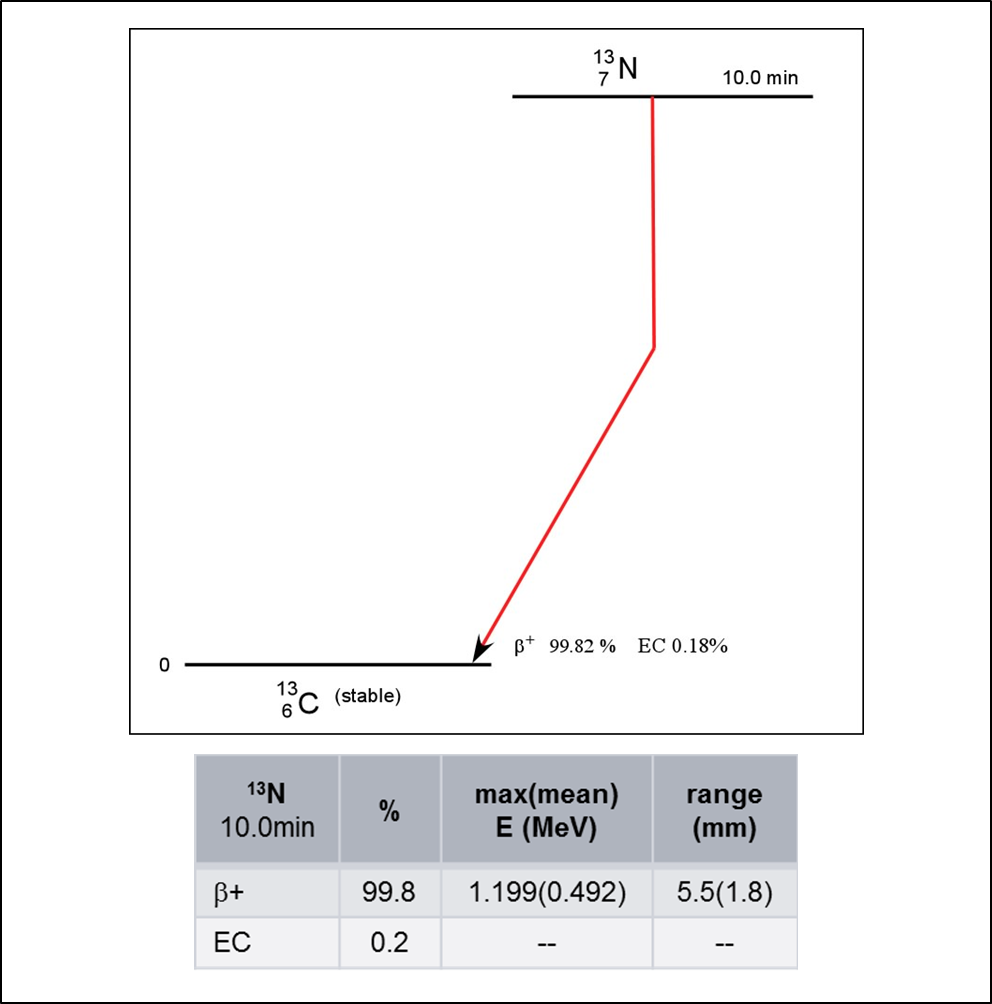


Figure A2: ^13^N


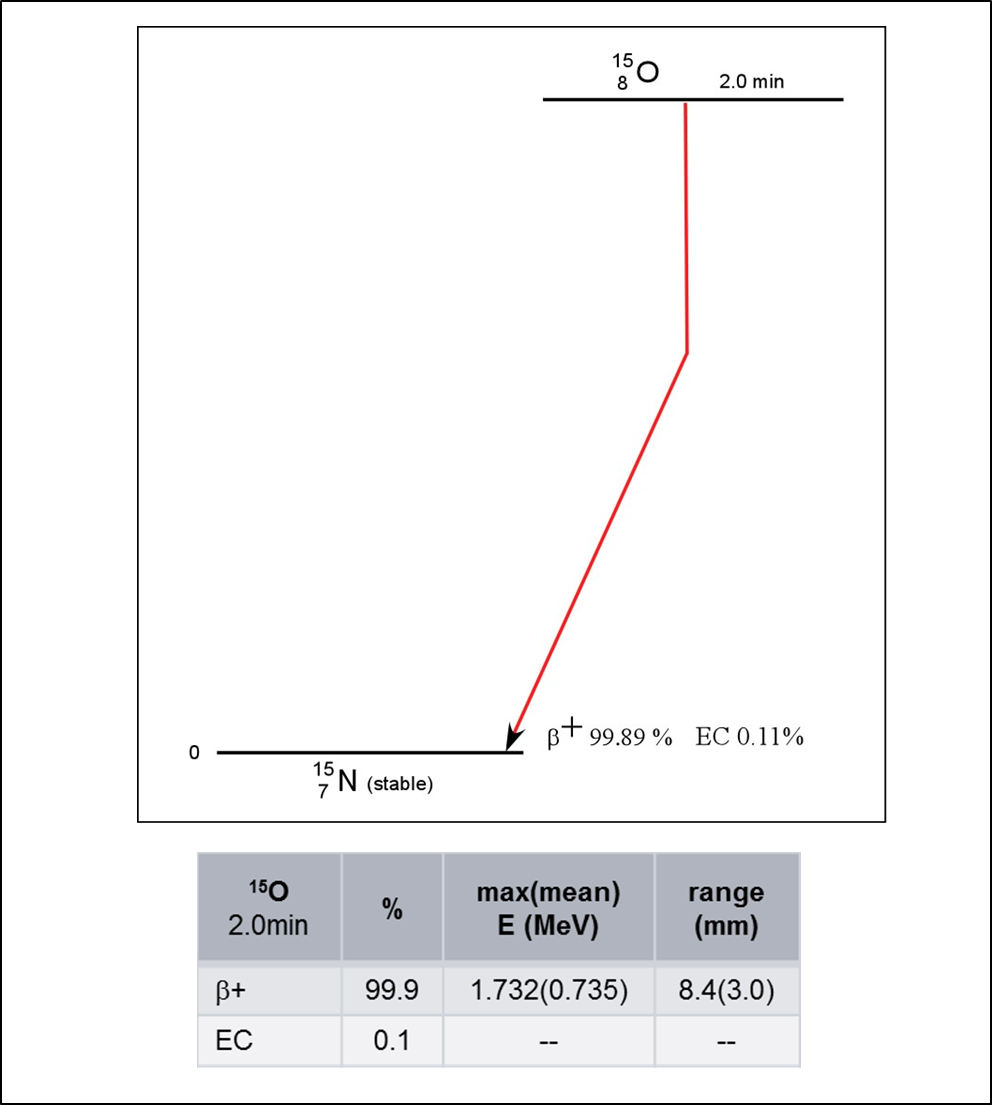


Figure A3: ^15^O


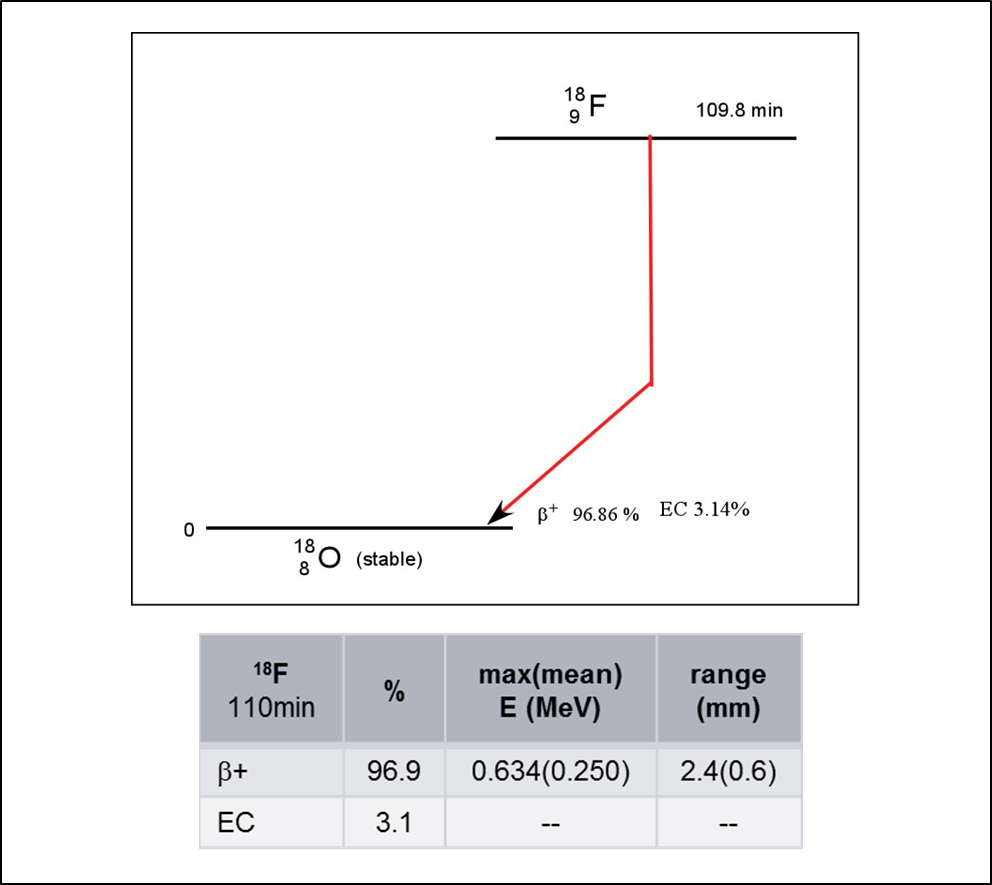


Figure A4: ^18^F


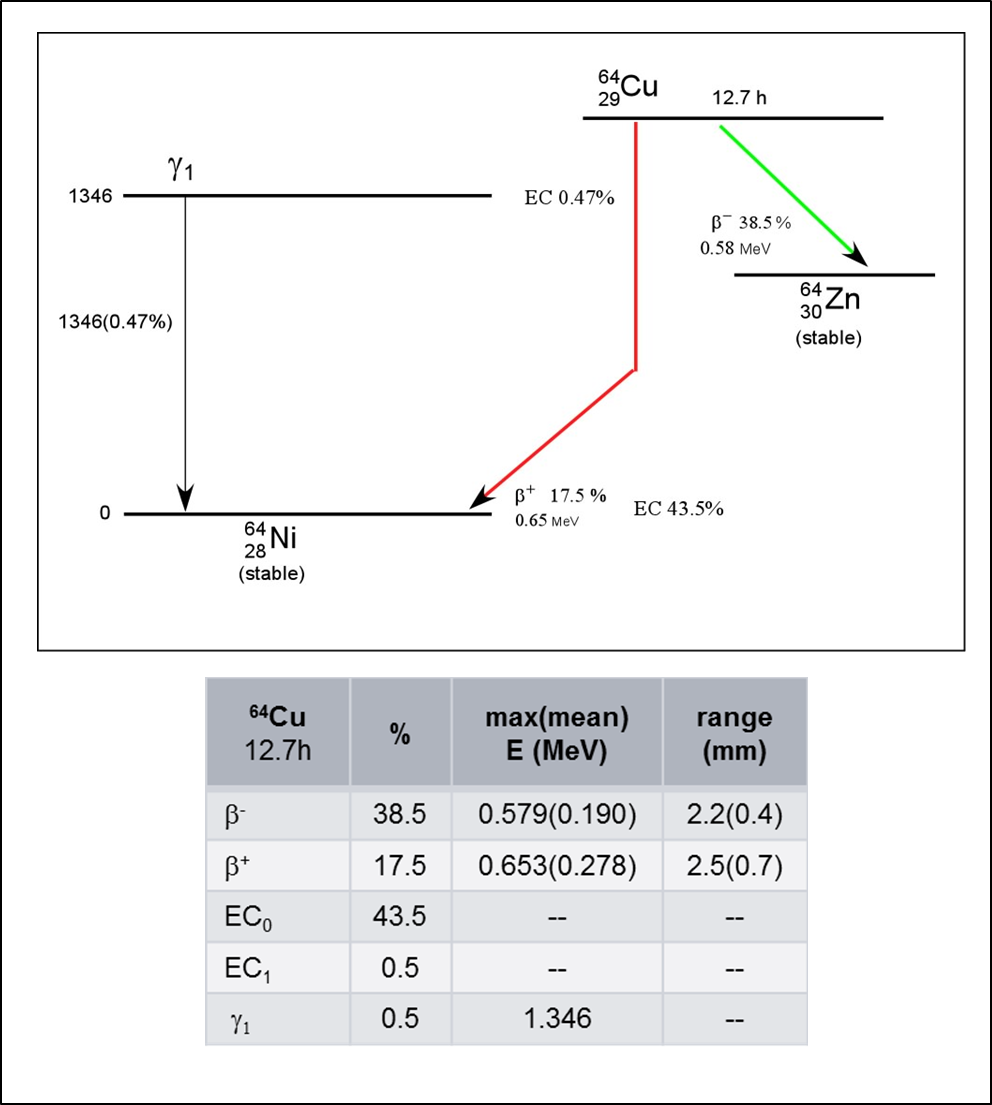


Figure A5: ^64^Cu


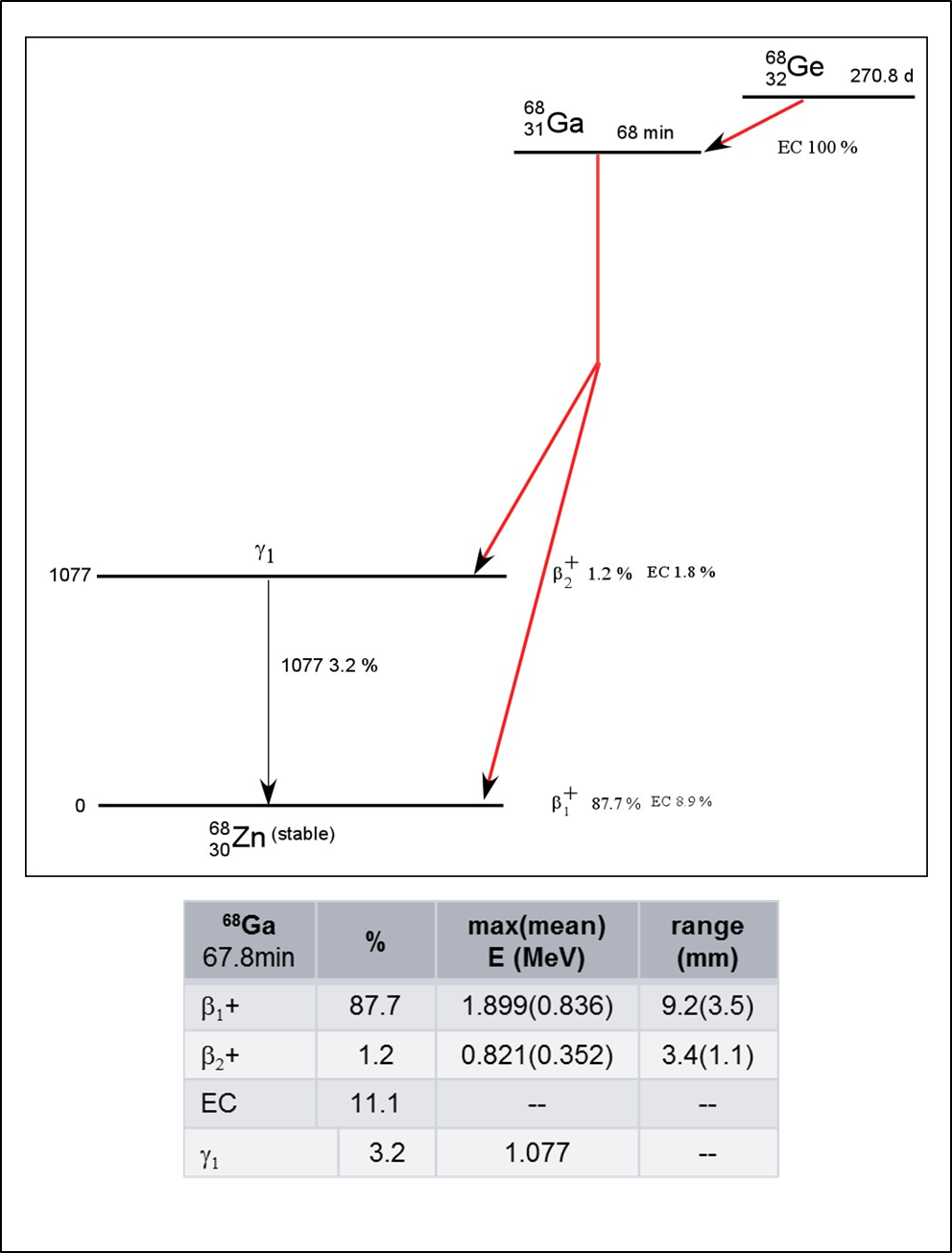


Figure A6: ^68^Ga


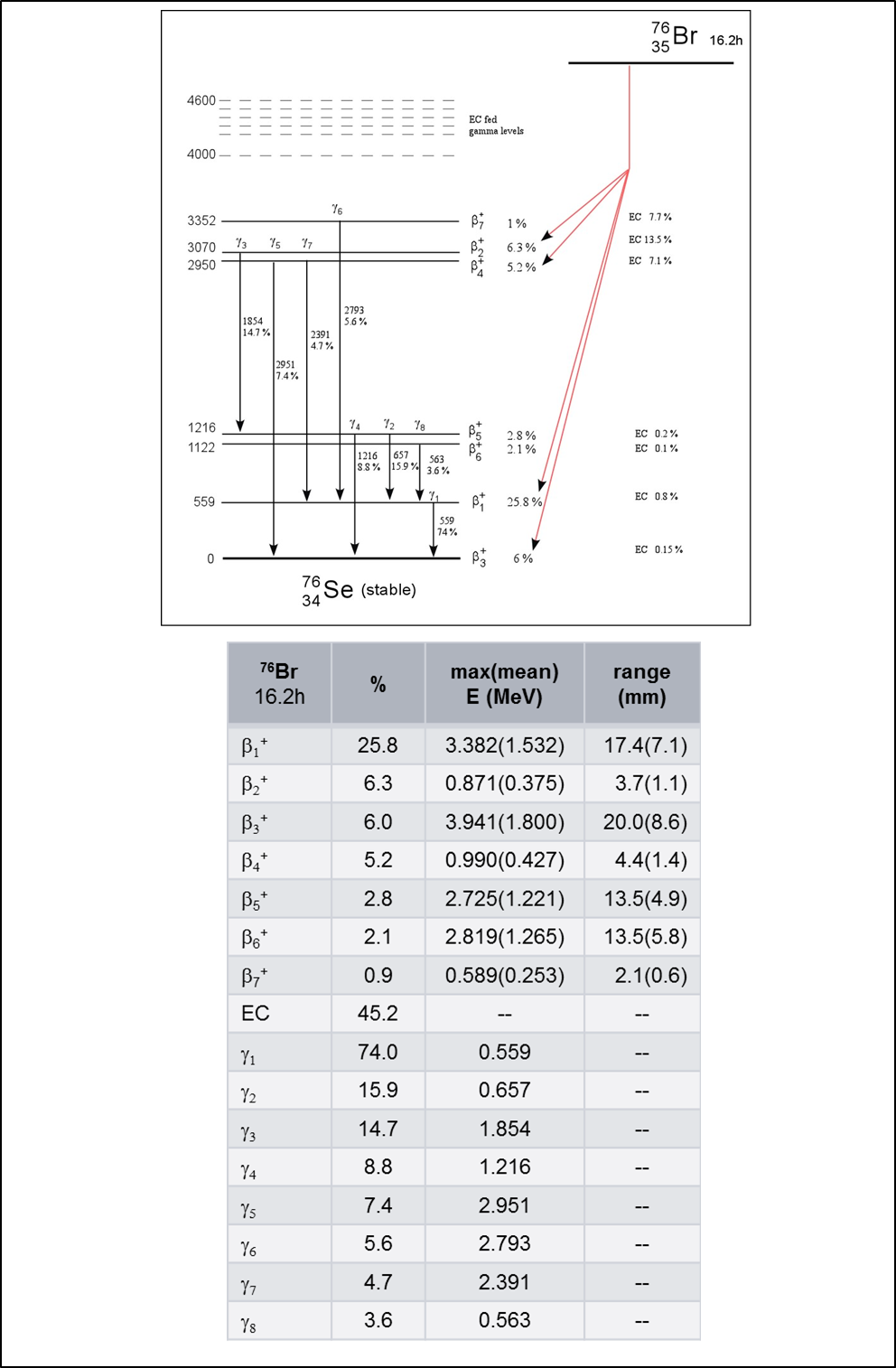


Figure A7: ^76^Br


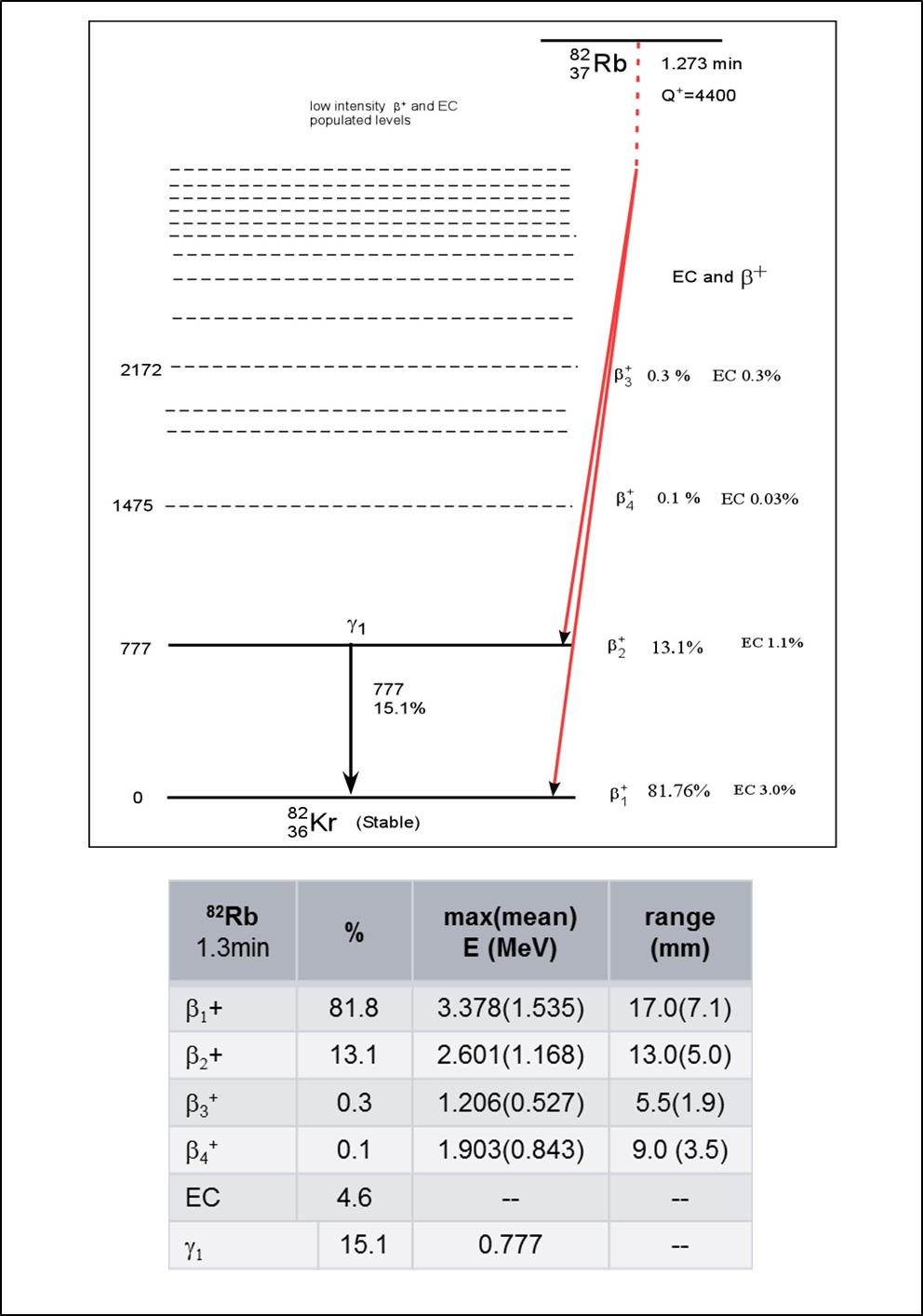
Figure A8: ^82^Rb


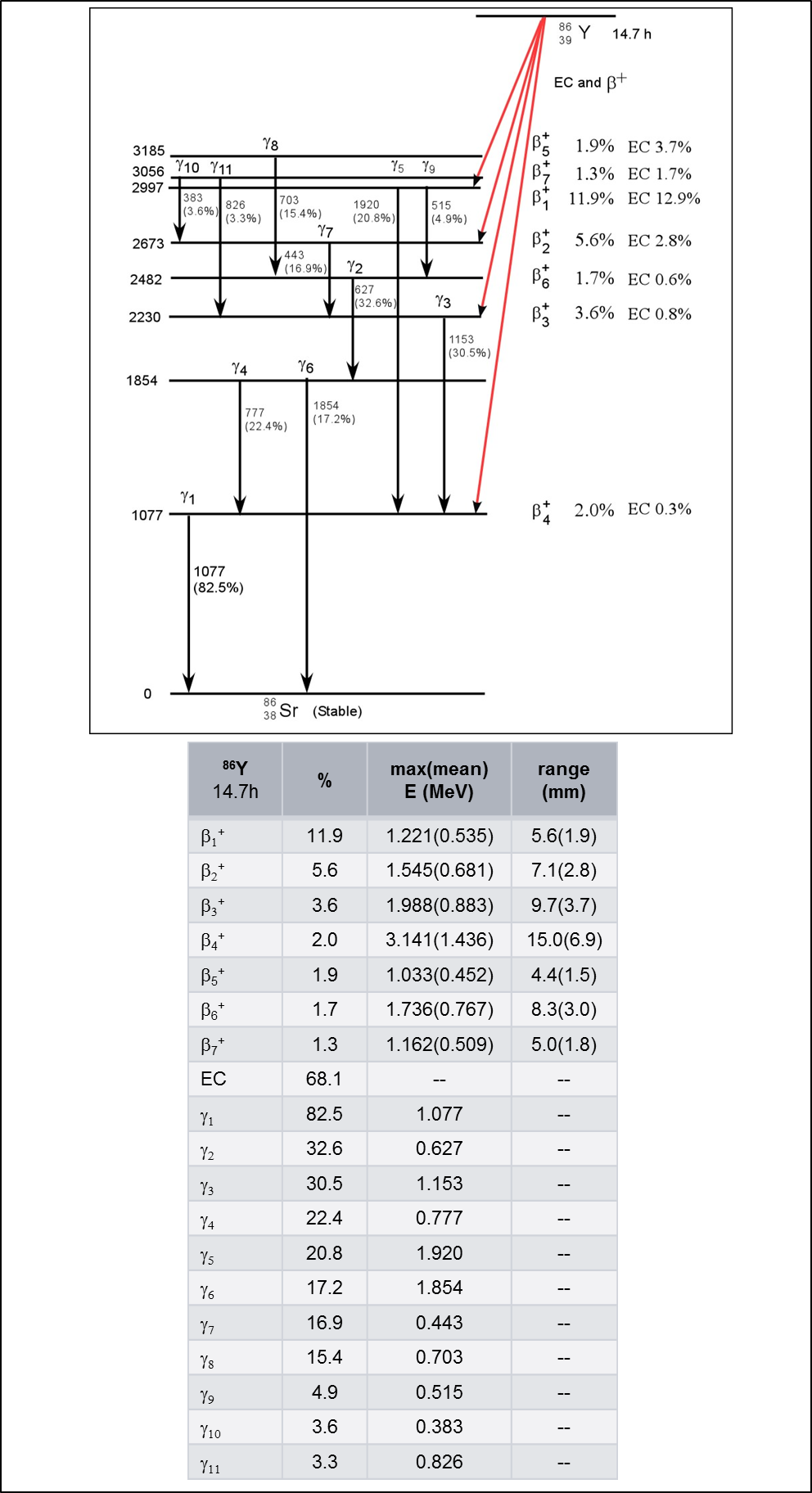


Figure A9: ^86^Y


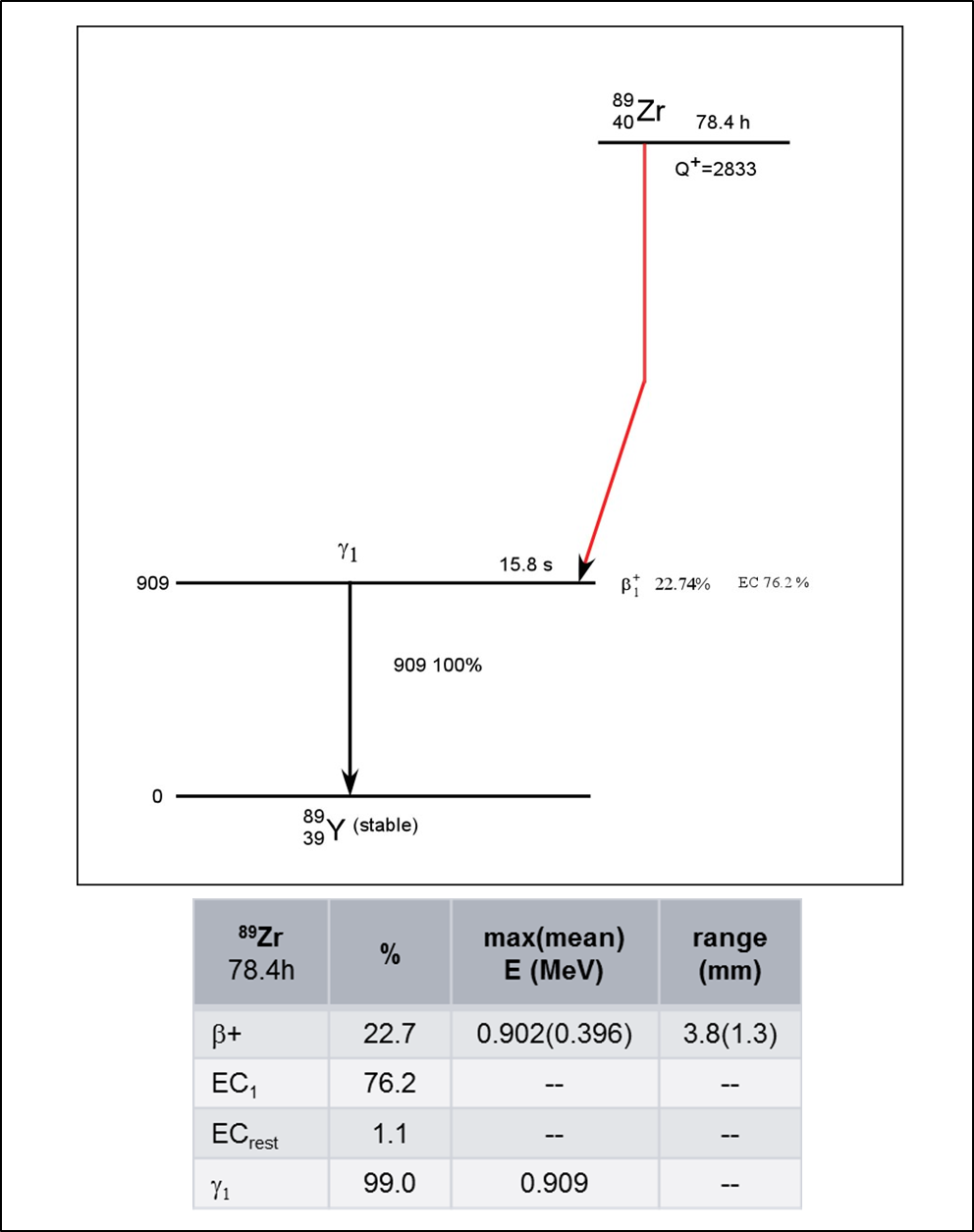


Figure A10: ^89^Zr


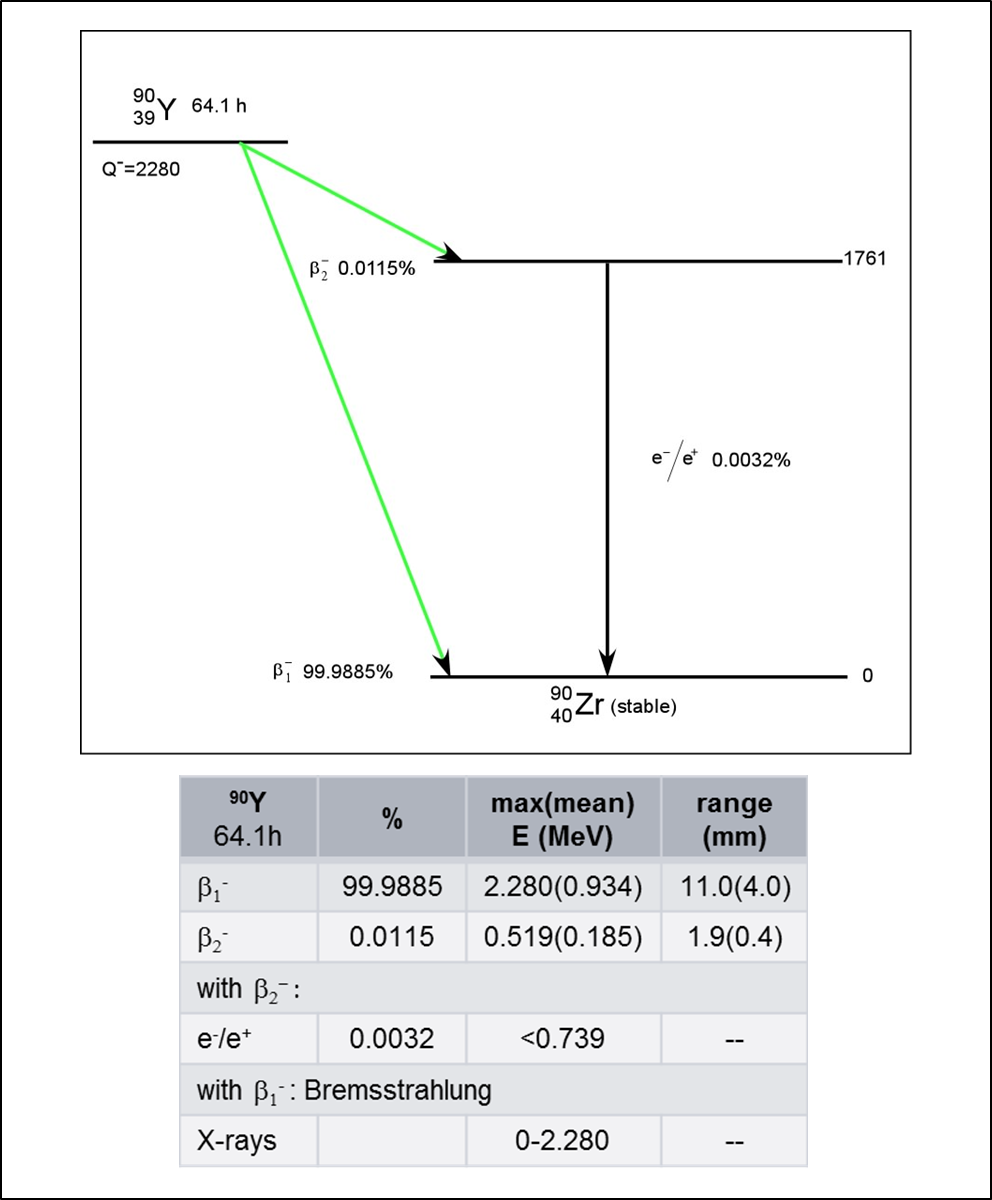


Figure A11: ^90^Y


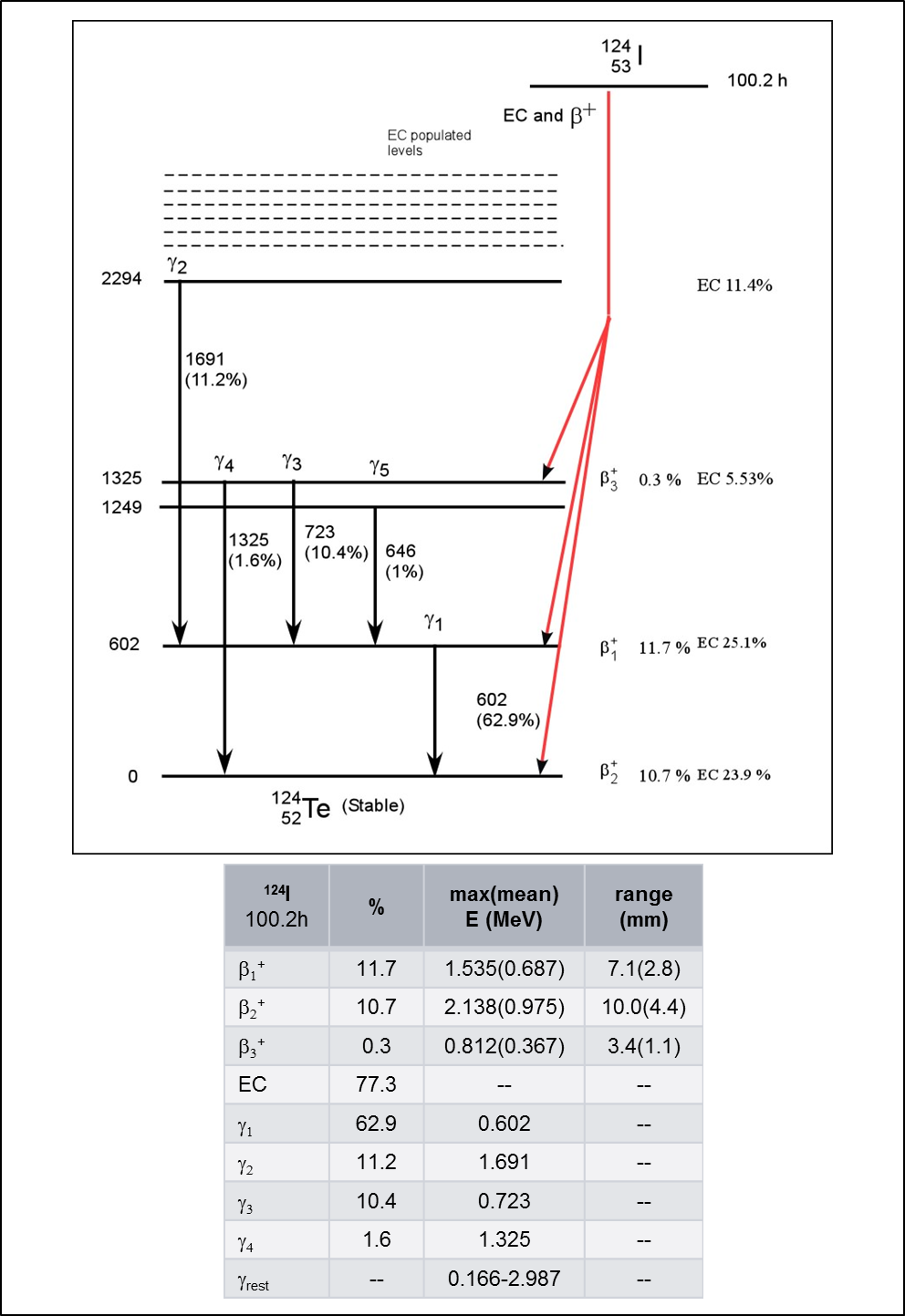


Figure A12: ^124^I
